# Glycaemic status and periodontitis severity Among patients attending a South African tertiary oral health centre: a prospective cross-sectional study

**DOI:** 10.3389/froh.2026.1974683

**Published:** 2026-10-28

**Authors:** L. Mukucha, S. Manenzhe, R. A. G. Khammissa, S. L. Shangase

**Affiliations:** 1 Periodontics and Oral Medicine, University of Pretoria, Pretoria, South Africa; 2 Department of Periodontology and Oral Medicine, Sefako Makgatho University, Pretoria, South Africa; 3 School of Dentistry, University of Witwatersrand, Johannesburg, South Africa

**Keywords:** diabetes mellitus, diabetes screening, hbA1c, oral health, periodontitis, prediabetes, South Africa

## Abstract

**Background:**

Diabetes mellitus (DM) and periodontitis are chronic inflammatory conditions with a well-established bidirectional relationship. Given the strong association between these conditions, individuals with periodontitis may have an increased likelihood of dysglycaemia that has not previously been recognised. This study aimed to determine the prevalence of diabetes and prediabetes among patients with periodontitis and to examine the relationship between glycaemic status and periodontitis severity.

**Aim:**

To determine the prevalence of DM and prediabetes among patients with periodontitis attending a tertiary oral health centre in South Africa and to examine the relationship between glycaemic status and periodontitis severity.

**Methods:**

A prospective cross-sectional study was conducted among 73 adults diagnosed with periodontitis at the University of Pretoria Oral Health Centre. Participants underwent comprehensive periodontal assessment and HbA1c testing. Previously diagnosed diabetes was recorded from the medical history. Among participants without a previous diagnosis, HbA1c ≥ 6.5% was considered a diabetic-range result for study screening purposes and HbA1c 5.7%–6.4% was considered the prediabetic range. Exact 95% confidence intervals (CIs) were calculated for prevalence estimates. HbA1c distributions were compared by periodontitis stage using the Kruskal–Wallis test. An exploratory multivariable logistic regression examined factors associated with dysglycaemia (diabetes or prediabetes).

**Results:**

Participants had a mean age of 50.7 years (SD 15.2); 47 (64.4%) were female. Generalised periodontitis was present in 69 (94.5%), with 46 (63.0%) classified as Stage 4 and 56 (76.7%) as Grade C. Thirteen participants had previously diagnosed diabetes. Among the 60 participants without a previous diagnosis, 6 (10.0%, 95% CI 3.8–20.5) had HbA1c values ≥6.5%, while 25 (41.7%, 95% CI 29.1–55.1) had HbA1c values of 5.7%–6.4%. Overall, 19/73 (26.0%) participants had either established diabetes or a diabetic-range HbA1c result, while 25/73 (34.2%) had HbA1c values in the prediabetic range. Median HbA1c was 5.7% (IQR 5.4%–6.1%). Stage 4 participants had a wider HbA1c range and a higher upper quartile than Stage 3 participants, but the difference was not statistically significant (*P* = 0.423). In exploratory regression analysis, increasing age was associated with dysglycaemia (OR 1.08, 95% CI 1.04–1.13; *P* < 0.001), while sex and Stage 4 status were not significantly associated.

**Conclusion:**

A substantial burden of dysglycaemia was identified among patients presenting with periodontitis, including diabetic-range HbA1c results among participants without previously diagnosed diabetes. These findings support consideration of appropriate glycaemic risk assessment and referral within periodontal care pathways. Larger multicentre studies, including periodontally healthy comparison groups, are required to further investigate this relationship and evaluate screening strategies.

## Introduction

Diabetes mellitus (DM) and periodontitis are common chronic diseases that interact through a complex bidirectional relationship. Hyperglycaemia is associated with altered host immune responses, increased inflammatory activity and impaired tissue repair, thereby increasing susceptibility to periodontal breakdown. Conversely, the chronic inflammatory burden associated with periodontitis may contribute to systemic inflammation and impaired glycaemic regulation. The relationship is therefore clinically relevant in both directions: diabetes is an important modifier of periodontal disease, while periodontal disease may represent an opportunity to identify individuals at increased metabolic risk (1–3).

The biological plausibility of this relationship is supported by several mechanisms. Persistent hyperglycaemia promotes formation of advanced glycation end-products and signalling through their receptors, alters neutrophil function and amplifies production of pro-inflammatory mediators. Within periodontal tissues, these changes can favour connective-tissue destruction and bone resorption. At the same time, periodontal inflammation can contribute to a systemic inflammatory milieu that may adversely influence insulin sensitivity and glycaemic control (2, 3).

The contemporary periodontal classification incorporates diabetes as an important modifier of periodontitis grading. Stage reflects severity and complexity, whereas grade incorporates evidence concerning progression and risk. Poorly controlled diabetes can influence grading because of its potential to accelerate periodontal destruction and adversely affect treatment response (4, 5). This makes periodontal clinics particularly relevant settings in which metabolic risk may be considered.

Although the association between periodontitis and diabetes is well established, the reverse clinical question—how frequently diabetes or prediabetes is present among people who present for periodontal care—has received comparatively less attention, especially in South Africa. Earlier epidemiological work has shown that diabetes is more frequently detected when glycaemic status is assessed objectively rather than by self-report, and systematic reviews have demonstrated higher HbA1c levels among people with periodontitis without known diabetes (6–9). More recent evidence continues to support the potential value of dental visits for identifying hyperglycaemia. A systematic review and meta-analysis found an association between periodontitis and HbA1c levels in people without diabetes, while clinical risk-model research has suggested that advanced periodontitis can contribute to identification of patients at increased diabetes risk (10, 11).

The potential screening role of the dental setting has become increasingly relevant because diabetes may remain undiagnosed for prolonged periods (12). Opportunistic screening therefore needs to be understood as part of a clinical pathway that includes appropriate testing, communication of results and referral rather than as a stand-alone diagnostic intervention.

The South African context is important. The burden of diabetes is substantial, while access to preventive and chronic disease services remains uneven. Tertiary oral health facilities may encounter patients who have limited access to routine medical screening and who present with advanced periodontal disease (13). In the present study, the cohort was drawn from a tertiary periodontal service and was characterised by a high proportion of advanced disease. Determining the prevalence of dysglycaemia in this clinical population may therefore provide useful evidence for developing integrated oral-medical screening pathways.

The primary aim of this study was to determine the prevalence of diabetes and prediabetes among adults diagnosed with periodontitis attending the University of Pretoria Oral Health Centre. Secondary objectives were to describe participant characteristics, quantify diabetic-range HbA1c and prediabetic-range HbA1c among participants without a previous diagnosis of diabetes, describe the stage and grade of periodontitis, and examine the relationship between HbA1c and periodontal disease severity.

## Materials and methods

### Study design and setting

A prospective cross-sectional study was conducted in the Department of Periodontics and Oral Medicine at the University of Pretoria Oral Health Centre (UPOHC), South Africa. Participants were recruited sequentially from patients attending the periodontics clinic until the target sample of 73 was reached.

### Participants

Adults diagnosed with periodontitis who consented to participation and HbA1c assessment were eligible. Patients who did not have periodontitis, declined participation or blood sampling, did not have complete periodontal charting, or reported tobacco smoking were excluded. Smoking status was based on participant report.

### Sample size

The primary outcome was the proportion of participants with an HbA1c value ≥6.5% (diabetic range for study screening purposes). Accordingly, a sample-size formula for a single proportion was used, with a Z statistic of 1.96 for a 95% confidence level, an expected prevalence of 22% based on the reported prevalence of diabetes in South Africa, and a precision (margin of error) of 5%. Because the study was conducted within a finite population of patients attending the Periodontics consulting rooms, a finite-population correction was applied.

Approximately 200 patients with periodontitis were seen per month across the periodontics ward, corresponding to an estimated maximum population of approximately 2,000 patients over 10 months. However, data collection was conducted by a single dentist, who was the primary investigator. The practically accessible population within the 10-month data-collection period was therefore estimated at a maximum of approximately 300 patients. For an accessible population of 300, the finite-population calculation yielded a sample size of 141 at 5% precision and 95% confidence. This number was not practically achievable because of budget constraints associated with the number of HbA1c tests required. The calculation was therefore also performed across different accessible population sizes. For an accessible population of 100, the calculated sample size at 5% precision and 95% confidence was 73. An accessible population of 100 was therefore selected for the study calculation, and 73 participants were ultimately recruited. The sample-size calculation was based on Naing et al. (2022).

### Periodontal assessment

A comprehensive periodontal examination was performed using a UNC-15 periodontal probe. Probing pocket depths were recorded at six sites per tooth. Gingival recession, plaque index, bleeding index and furcation involvement were also recorded. Periodontitis was classified according to the 2017 World Workshop classification framework. Staging incorporated disease severity and complexity, including radiographic bone loss according to the Stage I–IV framework. Grade was assigned using the relevant patient-related modifiers specified in the classification framework. Importantly, the HbA1c measurement obtained as part of the present study was not used to determine periodontal Grade. The examiner underwent intra- and inter-examiner calibration before data collection; agreement was 98% for intra-examiner calibration and 94% for inter-examiner calibration.

### Assessment of diabetes and glycaemic status

Participants provided information on previous diabetes diagnosis and type of diabetes. Approximately 2.5 mL of venous blood was collected into EDTA tubes and submitted to the Ampath laboratory for HbA1c analysis. Participants with a documented diabetes diagnosis who had an HbA1c result obtained within three months of periodontal examination were permitted to use that result rather than undergo repeat sampling.

For participants without a previous diagnosis of diabetes, HbA1c ≥ 6.5% was classified as a diabetic-range result for study screening purposes, while HbA1c 5.7%–6.4% was classified as the prediabetic range. A single HbA1c result in the diabetic range was not regarded as a definitive clinical diagnosis of diabetes without appropriate clinical confirmation. Overall, participants with previously diagnosed diabetes were reported separately from participants without a previous diagnosis who had diabetic-range HbA1c values.

### Statistical analysis

Data were analysed using Stata 16.1-compatible statistical definitions. Categorical variables are presented as frequencies and percentages; continuous variables are summarised using mean and standard deviation where appropriate and median with interquartile range for HbA1c because of its skewed distribution. Exact binomial 95% confidence intervals were calculated for diabetes, diabetic-range HbA1c and prediabetes estimates. The Kruskal–Wallis test was used to compare HbA1c distributions by periodontal stage.

As an exploratory analysis, a binary outcome of hyperglycaemia (diabetes or prediabetes) versus neither condition was modelled using logistic regression with age, sex and Stage 4 periodontitis as covariates. Given the small sample and observational design, this model was considered hypothesis-generating rather than confirmatory.

### Ethical considerations

Ethical approval was obtained from the University of Pretoria Research Ethics Committee (Ethics approval number 433/2023), and permission to conduct the study was obtained from the relevant clinical and institutional authorities. Written informed consent was obtained from participants. Participants were informed of their periodontal and glycaemic findings and those requiring further care were referred to the appropriate medical or dental practitioner.

## Results

### Participant characteristics

Seventy-three participants were included in the study. The mean age was 50.7 years (SD 15.2), with a median age of 53 years and a range of 20–75 years. Females comprised 47 (64.4%) participants, while 26 (35.6%) were male. (Table 1).

**Table 1:** Participant characteristics (*N* = 73).

Characteristic	*n* (%)
Age, years, mean (SD)	50.7 (15.2)
Female	47 (64.4)
Male	26 (35.6)

### Periodontal characteristics

Generalised periodontitis was recorded in 69 (94.5%) participants and localised disease in 4 (5.5%). No participant was classified as Stage 1 or Stage 2; 27 (37.0%) had Stage 3 and 46 (63.0%) had Stage 4 disease. One participant (1.4%) was Grade A, 16 (21.9%) Grade B and 56 (76.7%) Grade C. Thus, the cohort was predominantly characterised by advanced periodontal disease. (Table 2).

**Table 2:** Periodontal characteristics (*N* = 73).

Periodontal characteristic	*n* (%)
Generalised	69 (94.5)
Localised	4 (5.5)
Stage 3	27 (37.0)
Stage 4	46 (63.0)
Grade A	1 (1.4)
Grade B	16 (21.9)
Grade C	56 (76.7)

### Glycaemic status and previously unrecognised dysglycaemia

Thirteen participants (17.8%) reported a previous diagnosis of type 2 diabetes. Among the 60 participants without a previous diagnosis of diabetes, 6 (10.0%, 95% CI 3.8–20.5) had HbA1c values ≥6.5%, representing diabetic-range screening results, while 25 (41.7%, 95% CI 29.1–55.1) had HbA1c values of 5.7%–6.4%, consistent with the prediabetic range. The remaining 29 (48.3%) had HbA1c below 5.7%.

Across the full cohort, 13 participants had established diabetes and 6 additional participants without a previous diagnosis had diabetic-range HbA1c results. Thus, 19/73 (26.0%) participants had either established diabetes or a diabetic-range HbA1c result. Twenty-five participants had HbA1c values in the prediabetic range, representing 25/73 (34.2%) of the full cohort. The median HbA1c for the full cohort was 5.7% (IQR 5.4%–6.1%; range 4.9–10.6%). (Table 3).

**Table 3:** Overall glycaemic status in the study cohort (*N* = 73).

Glycaemic category	*n* (%)	95% CI
Established diabetes or diabetic-range HbA1c	19/73 (26.0)	16.5–37.6
Prediabetes	25/73 (34.2)	23.5–46.3
Neither diabetes nor prediabetes	29/73 (39.7)	28.5–51.9

Percentages are calculated using the full study cohort as the denominator. The 19 participants in the first category comprise 13 participants with established diabetes and 6 participants without a previous diagnosis who had HbA1c values ≥6.5%.

### Hba1c and periodontitis severity

Stage 3 participants had a median HbA1c of 5.7% (IQR 5.3%–6.0%; range 5.0%–7.8%), whereas Stage 4 participants had a median of 5.7% (IQR 5.4%–6.2%; range 4.9–10.6%). The broader range and higher upper quartile among Stage 4 participants indicate greater variability and the presence of higher HbA1c values in the most advanced disease group. However, the difference in HbA1c distributions between stages was not statistically significant (Kruskal–Wallis *P* = 0.423). (Table 4). Banjar et al. (18) also investigated the relationship between glycated haemoglobin levels and periodontitis stage in individuals without diabetes.

**Table 4:** Hba1c distribution by periodontitis stage.

Periodontitis stage	*n*	Median HbA1c % (IQR)	Range
Stage 3	27	5.7 (5.3–6.0)	5.0–7.8
Stage 4	46	5.7 (5.4–6.2)	4.9–10.6

### Exploratory analysis of dysglycaemia

In the exploratory multivariable model, 42 participants had hyperglycaemia (diabetes or prediabetes) and 31 had neither condition. Increasing age was independently associated with hyperglycaemia (OR 1.08 per one-year increase, 95% CI 1.04–1.13; *P* < 0.001). Female sex was not significantly associated with hyperglycaemia (OR 0.80, 95% CI 0.24–2.65; *P* = 0.713), nor was Stage 4 periodontitis (OR 1.98, 95% CI 0.61–6.37; *P* = 0.254). The wide confidence interval around the Stage 4 estimate reflects the limited sample size. These analyses should therefore be interpreted as exploratory and should not be used to infer causality.

## Discussion

The principal finding of this study was the substantial burden of dysglycaemia among patients presenting with periodontitis. Thirteen participants had established diabetes, and a further 6 participants without a previous diagnosis had HbA1c values in the diabetic range. In addition, more than two in five participants without previously diagnosed diabetes had HbA1c values consistent with prediabetes. These results indicate that periodontal services may encounter a clinically relevant burden of previously unrecognised metabolic risk.

The overall proportion of participants with established diabetes or a diabetic-range HbA1c result was 26.0%, compared with the approximately 22% population estimate used for the original sample-size calculation. Direct comparison should nevertheless be made cautiously because this was a selected tertiary periodontal cohort rather than a population-based sample. The high prevalence may partly reflect referral patterns and the advanced periodontal disease profile of the participants.

The finding that 6/60 (10.0%) participants without a previous diagnosis had HbA1c values in the diabetic range is particularly relevant from a screening perspective. These findings should be interpreted as screening results rather than definitive new diagnoses because confirmation through appropriate medical assessment may be required. This observation is consistent with evidence from dental settings demonstrating that opportunistic glycaemic screening can identify previously unrecognised dysglycaemia (17, 19, 20).

The prevalence of prediabetes is also clinically relevant. Among participants without a previous diagnosis of diabetes, 25/60 (41.7%) had HbA1c values in the prediabetic range. Identification of prediabetes provides an opportunity for risk communication and referral before overt diabetes is established. It also supports a broader interpretation of the dental visit: the periodontal consultation can function as a point of contact for identification of non-communicable disease risk, provided that appropriate referral and follow-up systems are in place.

The periodontal profile of this cohort is also noteworthy. Ninety-five percent had generalised disease, 63% had Stage 4 periodontitis and 77% had Grade C disease. This is not surprising in a tertiary periodontal service, but it is clinically relevant because the cohort represents patients with a high burden of periodontal destruction rather than the spectrum of disease encountered in primary dental care. The concentration of advanced disease may partly explain the high prevalence of dysglycaemia and limits the extent to which these findings can be extrapolated to patients with mild or early periodontitis.

Although Stage 4 participants had a wider HbA1c range and a higher upper quartile than Stage 3 participants, the difference was not statistically significant. The study should therefore not be interpreted as demonstrating a statistically confirmed relationship between periodontal stage and HbA1c. Rather, the data show a pattern of greater HbA1c variability in Stage 4 disease that warrants investigation in larger studies. Because of the small sample and narrow stage distribution, the present study cannot establish whether increasing periodontal severity independently predicts dysglycaemia.

The exploratory regression provides a further perspective. Age was associated with dysglycaemia, while Stage 4 disease was associated with approximately twice the odds of dysglycaemia but with a wide confidence interval that crossed the null value. The model was exploratory and hypothesis-generating, and the study was not designed to provide a stable multivariable assessment of periodontal stage. A larger multicentre study should prespecify a clinically informed model incorporating established diabetes-risk variables and periodontal severity.

The findings nevertheless have potential clinical implications for integrated periodontal and metabolic risk assessment. A periodontal clinic does not need to diagnose diabetes independently of the medical system in order to contribute to earlier detection. A pragmatic pathway could begin with a brief diabetes-risk assessment or review of known diabetes status, followed by HbA1c testing or referral according to local policy and available resources. Abnormal results should be communicated to the patient and referred for medical confirmation and management.

These findings also support coordinated referral and management pathways between oral health professionals and medical practitioners. Medical services should recognise periodontitis as an important comorbidity in patients with diabetes, while dental services should recognise periodontal presentation as a potential opportunity to identify dysglycaemia. Such bidirectional collaboration may facilitate more integrated management of oral and systemic health.

The choice of HbA1c is particularly relevant to dental practice because testing does not require fasting and provides an estimate of longer-term glycaemic exposure. (21) However, HbA1c should not be treated as infallible. Certain haematological conditions and altered red-cell turnover can influence results, and diagnostic confirmation may be required depending on the clinical context and applicable guidelines. The present study used laboratory venous HbA1c testing.

Evidence from recent systematic reviews indicates that periodontal therapy may improve glycaemic outcomes in people with diabetes, although the magnitude and certainty of this effect vary. Conversely, diabetes may influence periodontal treatment response, reinforcing the need for co-management rather than treating the two diseases as independent conditions (14–16).

An important strength of the study is its prospective data collection and objective laboratory assessment of HbA1c. Periodontal diagnosis was based on a comprehensive clinical examination and the examiner underwent calibration, with high intra- and inter-examiner agreement. The use of the contemporary staging and grading framework also permits the periodontal findings to be interpreted within a clinically meaningful disease-classification system.

The study has several limitations. First, the sample was small and drawn from a single tertiary institution; consequently, prevalence estimates have relatively wide confidence intervals and cannot be generalised to the South African population or to all dental patients. Second, the sample was enriched for advanced periodontitis, limiting comparison across the full spectrum of periodontal severity. Third, the cross-sectional design prevents conclusions about temporality or causality. Fourth, smoking status was self-reported and smoking was an exclusion criterion, which may introduce selection bias and limits generalisability to the broader periodontitis population. Fifth, some known diabetic participants used HbA1c results obtained within three months rather than a contemporaneous blood sample; this was clinically pragmatic but introduces some temporal variation in the laboratory measurement. Finally, the exploratory regression was limited by sample size and should be validated in a larger cohort.

An additional limitation is that the study did not include a periodontally healthy comparison group. Consequently, the findings cannot establish whether the observed distribution of HbA1c values differs from that of individuals without periodontitis. Future studies incorporating appropriate periodontally healthy controls would provide a stronger basis for evaluating the association between periodontal disease and glycaemic status.

Despite these limitations, the study provides locally relevant evidence for South Africa. The finding that 6/60 participants without a previous diagnosis had HbA1c values in the diabetic range, together with the high prevalence of prediabetes, suggests that periodontal services may offer a useful point of contact for identifying metabolic risk. The appropriate response is not necessarily universal laboratory testing of every dental patient, but development and evaluation of targeted screening pathways, particularly for patients with advanced periodontitis or other diabetes risk factors. Future research should use larger multicentre samples, include patients across the full spectrum of periodontal severity and periodontally healthy comparison groups, incorporate established diabetes-risk variables, and evaluate the cost-effectiveness and feasibility of screening and referral within South African oral healthcare services.

## Clinical implications

Periodontal clinics should consider a structured pathway for identifying patients with possible hyperglycaemia. Such a pathway could include recording diabetes history, identifying risk factors, considering HbA1c testing or referral in patients with advanced periodontitis, documenting abnormal findings and ensuring communication with the patient's medical practitioner.

Screening should be embedded within a referral system. A positive screening result should not be regarded as a definitive medical diagnosis without appropriate clinical confirmation where required. Similarly, identification of prediabetes should trigger risk communication and referral rather than simply being recorded in the dental record.

The findings also support bidirectional collaboration: medical services should recognise periodontitis as an important comorbidity in patients with diabetes, while dental services should recognise periodontal presentation as a potential opportunity to identify dysglycaemia. Coordinated diagnostic and treatment pathways between physicians and oral health professionals may support more integrated care.

## Conclusion

This prospective study identified a substantial burden of dysglycaemia among patients with periodontitis attending a South African tertiary oral health centre. Thirteen participants had established diabetes, while 6/60 (10.0%) participants without a previous diagnosis had HbA1c values in the diabetic range and 25/60 (41.7%) had values in the prediabetic range. Although Stage 4 periodontitis was associated with greater HbA1c variability, the difference in HbA1c distributions between Stage 3 and Stage 4 disease was not statistically significant. These findings support consideration of targeted glycaemic risk assessment and appropriate referral within periodontal care pathways, while larger multicentre studies including periodontally healthy comparison groups are needed to further investigate the relationship between periodontal disease and glycaemic status and to evaluate screening strategies.

## Strengths and limitations

Strengths include prospective recruitment, objective HbA1c measurement, comprehensive periodontal assessment, use of contemporary periodontal staging and grading, and examiner calibration with high agreement. Limitations include the small single-centre sample, predominance of advanced periodontitis, absence of a periodontally healthy comparison group, self-reported smoking status and exclusion of smokers, cross-sectional design, limited statistical power for multivariable modelling, and the use of some previously recorded HbA1c values among participants with established diabetes.

## Data Availability

The raw data supporting the conclusions of this article will be made available by the authors, without undue reservation.
